# ﻿Three novel species of *Helminthosporium* (Massarinaceae, Pleosporales) from China

**DOI:** 10.3897/mycokeys.94.95888

**Published:** 2022-12-21

**Authors:** Jingwen Liu, Yafen Hu, Xingxing Luo, Rafael F. Castañeda-Ruíz, Jian Ma

**Affiliations:** 1 College of Agronomy, Jiangxi Agricultural University, Nanchang, Jiangxi 330045, China Jiangxi Agricultural University Nanchang China; 2 Instituto de Investigaciones Fundamentales en Agricultura Tropical “Alejandro de Humboldt” (INIFAT), calle 1, esq. 2, Santiago de Las Vegas, Ciudad de La Habana, C. P. 17200, Cuba Instituto de Investigaciones Fundamentales en Agricultura Tropical “Alejandro de Humboldt” (INIFAT) Havana Cuba

**Keywords:** asexual Ascomycota, hyphomycetes, lignicolous fungi, phylogenetic analysis, taxonomy

## Abstract

Three new species of *Helminthosporium*, *H.nabanhensis*, *H.sinensis* and *H.yunnanensis* collected on dead branches of unidentified plants in Xishuangbanna, China, were proposed by morphological and molecular phylogenetic analysis. Phylogenetic analysis of the combined data of ITS-SSU-LSU-*TEF1*-*RPB2* sequences was performed using Maximum-Likelihood and Bayesian Inference, although *H.nabanhensis* and *H.sinensis* lack the *RPB2* sequences. Both molecular analyses and morphological data supported *H.nabanhensis*, *H.sinensis* and *H.yunnanensis* as three independent taxa within the Massarinaceae.

## ﻿Introduction

*Helminthosporium* Link was originally erected by Link (1809) with *H.velutinum* as the type species, and was mainly characterized by macronematous, cylindrical, septate conidiophores with polytretic conidiogenous cells that producing solitary (rarely in short chains), acropleurogenous, clavate or obclavate, distoseptate conidia with a flat, ringed pore at the base (Ellis 1961, 1971; Luttrell 1964; Seifert et al. 2011). The genus became a repository for a large amount of species due to a lack of understanding of the generic concepts. To date, about 770 epithets for *Helminthosporium* are listed in Index Fungorum (2022), but most of these were not congeneric with the generic type in development of conidia and conidiophores. Ellis (1961) provided a review on *Helminthosporium*, and accepted ten species. Luttrell (1963, 1964) examined the type species and defined the generic concept, and Sivanesan (1987) transferred several unrelated pathogens of the *Poaceae* from *Helminthosporium* to the genera *Cochliobolus* (anamorph *Bipolaris*), *Setosphaeria* (anamorph *Exserohilum*) and *Pyrenophora* (anamorph *Drechslera*). Siboe et al. (1999) subsequently provided a synoptic table of the main morphological features that distinguish 27 accepted *Helminthosporium* species. Since then, 27 additional species have been described in the genus (Zhang et al. 2004, 2007, 2010; Shirouzu and Harada 2008; Zhang and Zhang 2009; Zhang and Sun 2010; Zhao and Zhao 2012; Wang et al. 2014; Tanaka et al. 2015; Zhu et al. 2016; Alves-Barbosa et al. 2017; Tian et al. 2017; Crous et al. 2018, 2019; Zhao et al. 2018; Boonmee et al. 2021; Chen et al. 2022). Voglmayr and Jaklitsch (2017) revealed the phylogenetic relationships of *Corynespora*, *Exosporium* and *Helminthosporium* species, synonymized *Exosporium* with *Helminthosporium*, and confirmed 17 species in *Helminthosporium* by morphological and molecular systematic analysis, but the generic concept has been widened by adding four *Corynespora* species that produce terminal, monotretic conidiogenous cells. So it is challenging to classify *Corynespora* and *Helminthosporium* species based on morphology alone because the distinction between monotretic vs. polytretic conidiogenous cells is the only character for separating *Corynespora* and *Helminthosporium*. Based on the records of Species Fungorum 2021, Konta et al. (2021) summarized the morphology, host information, locality, sequence data and related references of 216 *Helminthosporium* species reported worldwide. Unfortunately, sequence data for most species are unavailable, and only 27 species are represented by the DNA sequence in GenBank (Chen et al. 2022).

*Helminthosporium* is worldwide in distribution, usually found as a common saprobe on leaf or twig litter, but one specie, *H.solani*, is an economically important pathogen causing silver scurf disease in potatoes worldwide (Alcorn 1983; Voglmayr and Jaklitsch 2017; Boonmee et al. 2021). To date, only 28 species have been recorded in China, viz. *H.aquaticum*, *H.bambusicola*, *H.cantonense*, *H.chengduense*, *H.chinense*, *H.citri*, *H.conidiophorellum*, *H.constrictum*, *H.corchori*, *H.dongxingense*, *H.guangxiense*, *H.hunanense*, *H.ipomoeae*, *H.juglandis*, *H.lablab*, *H.ligustri*, *H.marantae*, *H.multiseptatum*, *H.nanjingense*, *H.obpyriforme*, *H.oplismeni*, *H.ovoideum*, *H.piperis*, *H.pseudomicrosorum*, *H.rhodomyrti*, *H.sichuanense*, *H.subhyalinum* and *H.submersum* (Zhang et al. 2004; Zhang and Zhang 2009; Zhang et al. 2010; Zhang and Sun 2010; Zhao and Zhao 2012; Wang et al. 2014; Zhu et al. 2016; Zhao et al. 2018; Chen et al. 2022).

Xishuangbanna lies on the northern edge of tropical Southeast Asia. It is located in the southwestern part of Yunnan Province, China. It covers 19,125 km^2^ and has a mountainous topography and humid tropical monsoon climate, with an average annual temperature of 19.3–23.9 °C, and an average annual precipitation of 1200–1800 mm. The primary forest vegetation types are tropical seasonal rain forest, tropical montane rain forest, evergreen broad-leaved forest, monsoon forest over limestone, and monsoon forest on river banks (Cao and Zhang 1997). Such conditions create a very wide range of habitats favoring the growth of various microbial species. During our continuing mycological surveys of saprobic microfungi from plant debris in this region, three interesting hyphomycetes with morphological features typical of *Helminthosporium* were collected on dead branches. Based on morphological data and multi-locus phylogenetic analysis, they were described as new to science in the present study.

## ﻿Materials and methods

### ﻿Sample collection, isolation and morphological studies

Samples of dead branches were collected from humid environments and river banks in the forest ecosystems of Xishuangbanna, Yunnan Province, China, and returned to the laboratory in Ziploc bags. Samples were processed and examined following the methods described in Ma et al. (2011). Fungi were mounted in a drop of lactic acid on microscope slides, and examined and photographed with an Olympus microscope (model BX 53), with a 100 × (oil immersion) objective at the same background color and scale. Adobe Photoshop 7.0 was used for image processing to assemble photographs into images. Single-spore isolations were made on potato dextrose agar (PDA) following Goh (1999). Colony colors were assessed according to the charts of Rayner (1970). All fungal strains were stored in 10% sterilized glycerin at 4 °C for further studies. The studied specimens and cultures were deposited in the Herbarium of Jiangxi Agricultural University, Plant Pathology, Nanchang, China (**HJAUP**).

### ﻿DNA extraction, PCR amplification and sequencing

Genomic DNA was extracted from fungal mycelia grown on PDA, using the Solarbio Fungi Genomic DNA Extraction Kit following the manufacturer’s protocol (Solarbio, China). The DNA amplification was performed by polymerase chain reaction (PCR) using the respective loci (ITS, SSU, LSU, *TEF1*, *RPB2*). Primer sets used for these genes were as follows: ITS: ITS5/ITS4 (White et al. 1990), SSU: 18S-F/18S-R, LSU: 28S1-F/28S3-R (Xia et al. 2017), *TEF1*: EF1-983F/EF1-2218R (Rehner 2001; Zhao et al. 2018) and *RPB2*: dRPB2-5f/dRPB2-7r (Voglmayr et al. 2016). The final volume of the PCR reaction was 25 μl, containing 1 μl of DNA template, 1 μl each of the forward and reverse primer, 12.5 μl of 2 × Power Taq PCR MasterMix and 9.5 μl of double-distilled water (ddH_2_O). The PCR thermal cycling conditions of ITS, SSU and LSU were initialized at 94 °C for 3 min, followed by 35 cycles of denaturation at 94 °C for 30 s, annealing at 55 °C for 50 s, elongation at 72 °C for 1 min, a final extension at 72 °C for 10 min, and finally kept at 4 °C, the *TEF1* and *RPB2* were initialized at 95 °C for 3 min, followed by 35 cycles of denaturation at 95 °C for 30 s, annealing at a suitable temperature for 30 s, elongation at 72 °C for 1 min, a final extension at 72 °C for 10 min, and finally kept at 4 °C. Annealing temperature was 60 °C for *TEF1*, 56 °C for *RPB2*. The PCR products were checked on 1% agarose gel electrophoresis stained with ethidium bromide. Purification and DNA sequencing were carried out at Beijing Tsingke Biotechnology Co., Ltd. China.

### ﻿Sequence alignment and phylogenetic analysis

The newly generated sequences together with other sequences obtained from GenBank (Table 1) were initially aligned using MAFFTv.7 (Katoh and Standley 2013) on the online server (http://maffTh.cbrc.jp/alignment/server/), and optimized manually when needed. To establish the identity of the isolates at species level, phylogenetic analyses were conducted first individually for each locus and then as combined analyses of five gene loci (ITS, LSU, SSU, *TEF1* and *RPB2*). Five aligned data sets of ITS, LSU, SSU, *TEF1* and *RPB2* are concatenated using the concatenated sequence function of Phylosuite software v1.2.1 (Zhang et al. 2020a), and absent sequence data (i.e., ITS, LSU, SSU, *TEF1* and *RPB2* sequence data) in the alignments were treated with the question mark as missing data. Phylosuite software v1.2.1 (Zhang et al. 2020a) was used to construct the phylogenetic tree based on ITS, SSU, LSU, *TEF1* and *RPB2* sequence data. The concatenated aligned dataset was analyzed separately using Maximum likelihood (ML) and Bayesian inference (BI). Maximum likelihood phylogenies were inferred using IQ-TREE (Nguyen et al. 2015) under Edge-linked partition model for 10000 ultrafast bootstraps (Hoang et al. 2017). The final tree was selected among suboptimal trees from each run by comparing the likelihood scores using the TIM2e+I+G4 for ITS+*RBP2*, TVMe+I+G4 for LSU+SSU, and TNe+R2 for *TEF1* substitution model. Bayesian Inference phylogenies were inferred using MrBayes 3.2.6 (Ronquist et al. 2012) under partition model (2 parallel runs, 2000000 generations), in which the initial 25% of sampled data were discarded as burn-in. The best-fit model was SYM+I+G4 for ITS+*RBP2*, LSU+SSU; SYM+G4 for *TEF1*. ModelFinder (Kalyaanamoorthy et al. 2017) was used to select the best-fit partition model (Edge-linked) using BIC criterion. The trees were viewed in FigTree v. 1.4.4 (http://tree.bio.ed.ac.uk/software/figtree) and further edited in Adobe Illustrator 2021.

**Table 1.** Species and GenBank accession numbers of DNA sequences used in this study. New sequences are in bold.

﻿ Taxon	Strain	Genbank accession numbers				
		SSU	LSU	ITS	﻿*RPB2*	*TEF1*
﻿*Byssotheciumcircinans*	CBS 675.92	GU205235	GU205217	OM337536	﻿DQ767646	GU349061
*Corynesporacassiicola*	CBS 100822	GU296144	GU301808	–	﻿GU371742	GU349052
*Corynesporasmithii*	L120	–	KY984297	KY984297	﻿KY984361	KY984435
*Corynesporasmithii*	L130	KY984419	KY984298	KY984298	﻿KY984362	KY984436
*Cyclothyriellarubronotata*	TR, CBS 121892	–	KX650541	KX650541	﻿KX650571	KX650516
*Cyclothyriellarubronotata*	TR9, CBS 141486	KX650507	KX650544	KX650544	﻿KX650574	KX650519
*Helminthosporiumaquaticum*	MFLUCC 15-0357, S-096^HT^	KU697310	KU697306	KU697302	–	–
*Helminthosporiumaustriacum*	L132 ^HT^, CBS 139924	KY984420	KY984301	KY984301	﻿KY984365	KY984437
*Helminthosporiumaustriacum*	L137	–	KY984302	KY984302	﻿KY984366	KY984438
*Helminthosporiumaustriacum*	L169, CBS 142388	–	KY984303	KY984303	﻿KY984367	KY984439
*Helminthosporiumcaespitosum*	L141	–	KY984305	KY984305	﻿KY984368	–
*Helminthosporiumcaespitosum*	L151	–	KY984306	KY984306	﻿KY984369	–
*Helminthosporiumcaespitosum*	L99 ^HT^, CBS 484.77	KY984421	JQ044448	JQ044429	KY984370	KY984440
﻿*Helminthosporiumchengduense*	UESTC 22.0024, CGMCC 3.23575 ^HT^	ON557757	ON557745	ON557751	﻿ON563073	ON600598
*Helminthosporiumchengduense*	UESTC 22.0025	ON557756	ON557744	ON557750	﻿ON563072	ON600597
*Helminthosporiumchiangraiense*	MFLUCC 21-0087 ^HT^	–	MZ538538	MZ538504	–	–
﻿*Helminthosporiumchinense*	UESTCC 22.0026, CGMCC 3.23570 ^HT^	ON557760	ON557748	ON557754	﻿–	ON600601
﻿*Helminthosporiumchlorophorae*	BRIP 14521	–	–	AF120259	–	–
*Helminthosporiumdalbergiae*	H 4628, MAFF 243853	AB797231	AB807521	LC014555	﻿–	AB808497
*Helminthosporiumendiandrae*	CBS 138902, CPC 22194 ^HT^	–	KP004478	KP004450	–	–
﻿*Helminthosporiumerythrinicola*	CBS 145569 ^HT^	–	MK876432	NR_165563	﻿MK876486	–
*Helminthosporiumgenistae*	L128, CBS 139921	KY984422	KY984308	KY984308	﻿KY984372	–
*Helminthosporiumgenistae*	L129, CBS 139922	KY984423	KY984309	KY984309	﻿KY984373	–
*Helminthosporiumgenistae*	L142 ^ET^, CBS 142597	–	KY984310	KY984310	﻿KY984374	–
*Helminthosporiumhispanicum*	L109 ^HT^, CBS 136917	KY984424	KY984318	KY984318	﻿KY984381	KY984441
*Helminthosporiumitalicum*	MFLUCC 17-0241	–	KY815015	KY797638	–	KY815021
*Helminthosporiumjuglandinum*	L118 ^HT^, CBS 136922	–	KY984321	KY984321	﻿KY984384	KY984444
*Helminthosporiumjuglandinum*	L97, CBS 136911	KY984425	KY984322	KY984322	﻿KY984385	KY984445
*Helminthosporiumleucadendri*	CBS 135133, CPC 19345 ^HT^	–	KF251654	KF251150	﻿KF252159	KF253110
﻿*Helminthosporiumlivistonae*	CPC 32158, CBS 144413 ^HT^	–	NG_064539	NR_160348	–	–
*Helminthosporiummagnisporum*	H 4627, MAFF 239278, TS 33 ^HT^	AB797232	AB807522	AB811452	﻿–	AB808498
*Helminthosporiummassarinum*	KT 1564 ^HT^, CBS 139690	AB797234	AB807524	AB809629	﻿–	AB808500
*Helminthosporiummassarinum*	KT 838^EP^, MAFF 239604	AB797233	AB807523	AB809628	﻿–	AB808499
*Helminthosporiummicrosorum*	L94	KY984426	KY984327	KY984327	﻿KY984388	KY984446
*Helminthosporiummicrosorum*	L95	–	KY984328	KY984328	﻿KY984389	KY984447
*Helminthosporiummicrosorum*	L96 ^ET^, CBS 136910	KY984427	KY984329	KY984329	﻿KY984390	KY984448
***Helminthosporiumnabanhensis***	**HJAUP C2054** ^ET^	**OP555400**	**OP555398**	**OP555394**	–	**OP961931**
﻿*Helminthosporiumnanjingensis*	ZM020380	–	–	KF192322	–	–
*Helminthosporiumoligosporum*	L106	–	KY984330	KY984330	﻿KY984391	KY984449
*Helminthosporiumoligosporum*	L92, CBS 136908	KY984428	KY984332	KY984332	﻿KY984393	KY984450
*Helminthosporiumoligosporum*	L93^ET^, CBS 136909	–	KY984333	KY984333	﻿KY984394	KY984451
*Helminthosporiumquercinum*	L90 ^HT^, CBS 136921	KY984429	KY984339	KY984339	﻿KY984400	KY984453
*Helminthosporiumquercinum*	L91	–	KY984340	KY984340	﻿KY984401	KY984454
***Helminthosporiumsinensis***	**HJAUP C2121** ^ET^	**OP555399**	**OP555397**	**OP555393**	–	**OP961932**
*Helminthosporiumsolani*	CBS 365.75	KY984430	KY984341	KY984341	﻿KY984402	KY984455
*Helminthosporiumsolani*	CBS 640.85	–	KY984342	KY984342	﻿KY984403	–
*Helminthosporiumsubmersum*	﻿UESTCC 22.0021	ON557759	ON557747	ON557753	ON563075	ON600600
*Helminthosporiumsubmersum*	﻿MFLUCC 16-1360 ^HT^	MG098796	MG098787	–	–	MG098586
*Helminthosporiumsubmersum*	MFLUCC 16-1290^PT^	MG098797	MG098788	MG098780	﻿MG098592	MG098587
﻿*Helminthosporiumsyzygii*	CBS 145570 ^HT^	–	MK876433	NR_165564	﻿MK876487	–
*Helminthosporiumtiliae*	L171	–	KY984343	KY984343	﻿KY984404	KY984456
*Helminthosporiumtiliae*	L88 ^ET^, CBS 136907	KY984431	KY984345	KY984345	﻿KY984406	KY984457
*Helminthosporiumtiliae*	L89	–	KY984346	KY984346	﻿KY984407	–
*Helminthosporiumvelutinum*	H 4626, MAFF 243854	AB797240	AB807530	LC014556	﻿–	AB808505
*Helminthosporiumvelutinum*	H 4739, MAFF 243855	AB797235	AB807525	LC014557	﻿–	AB808501
*Helminthosporiumvelutinum*	L115, CBS 136924	–	KY984347	KY984347	﻿KY984408	KY984458
*Helminthosporiumvelutinum*	L116	–	KY984348	KY984348	﻿KY984409	KY984459
*Helminthosporiumvelutinum*	L117	–	KY984349	KY984349	﻿KY984410	KY984460
*Helminthosporiumvelutinum*	L126	–	KY984350	KY984350	﻿KY984411	KY984461
*Helminthosporiumvelutinum*	L127	–	KY984351	KY984351	﻿KY984412	KY984462
*Helminthosporiumvelutinum*	L131 ^ET^, CBS 139923	KY984432	KY984352	KY984352	﻿KY984413	KY984463
*Helminthosporiumvelutinum*	L98	KY984433	KY984359	KY984359	﻿KY984417	KY984466
*Helminthosporiumvelutinum*	yone 96, MAFF 243859	AB797239	AB807529	LC014558	﻿–	AB808504
***Helminthosporiumyunnanensis***	**HJAUP C2071** ^ET^	**OP555392**	**OP555396**	**OP555395**	**OP961934**	**OP961933**
*Massarinacisti*	CBS 266.62, JCM 14140 ^HT^	AB797249	AB807539	LC014568	﻿FJ795464	AB808514
*Massarinaeburnea*	CBS 473.64	AF164367	GU301840	AF383959	﻿GU371732	GU349040
*Massarinaeburnea*	H 3953, CBS 139697	AB521718	AB521735	LC014569	﻿–	AB808517
*Periconiabyssoides*	H 4600, MAFF 243872	AB797280	AB807570	LC014581	﻿–	AB808546
*Periconiadigitata*	CBS 510.77	AB797271	AB807561	LC014584	﻿–	AB808537
*Periconiapseudodigitata*	KT 1395, CBS 139699, MAFF 239676 ^HT^	﻿NG_064850	NG_059396	NR_153490	﻿–	AB808540
﻿*Pseudosplanchnonemaphorcioides*	L16, CBS 122935	KY984434	KY984360	KY984360	﻿KY984418	KY984467
*Stagonosporapaludosa*	CBS 135088, S601^NT^	–	KF251760	KF251257	﻿KF252262	KF253207
*Stagonosporaperfecta*	KT 1726A, MAFF 239609	AB797289	AB807579	AB809642	﻿–	AB808555
*Stagonosporapseudoperfecta*	KT 889, CBS 120236, MAFF 239607 ^HT^	AB797287	AB807577	AB809641	﻿–	AB808553
*Stagonosporatainanensis*	KT 1866, MAFF 243860	AB797290	AB807580	AB809643	﻿–	AB808556

^1^“–”, sequence is unavailable. ^2^Strain with ET (epitype), HT (holotype), NT (neotype), and PT (paratype). ^3^Abbreviations: **CBS**: Central Bureau voor Schimmel cultures, Utrecht, The Netherlands; **CGMCC**: China General Microbiological Culture Collection Center; **CPC**: Collection of Pedro Crous housed at CBS; **HJAUP**: Herbarium of Jiangxi Agricultural University, Plant Pathology; **MAFF**: the National Institute of Agrobiological Sciences, Japan; **MFLUCC**: Mae Fah Luang University Culture Collection, Chiang Rai, Thailand; **UESTCC**: The University of Electronic Science and Technology Culture Collection, Chengdu, China; **ITS**: Internal Transcribed Spacer; **SSU**: Small Subunit Ribosomal; **LSU**: Large Subunit Ribosomal; ***TEF1***: Transcriptional Enhancer Factor 1-alpha; ***RPB2***: The Second Largest Subunit of RNA Polymerase II; others are not registered abbreviations.

## ﻿Results

### ﻿Molecular phylogeny

**Figure 1.**
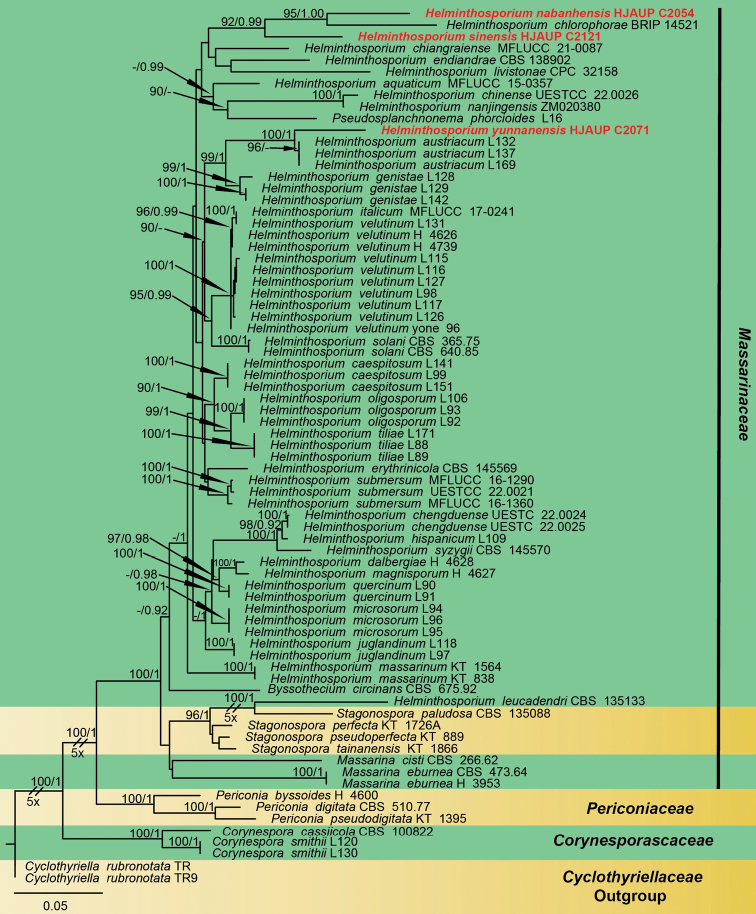
Phylogram of Massarinaceae based on combined ITS, SSU, LSU, *RPB*2 and *TEF*1 sequences. The ML and BI bootstrap support values above 90% and 0.90 are shown at the first and second position, respectively. The tree is rooted to *Cyclothyriellarubronotata* (TR) and *C.rubronotata* (TR9). Strains from the current study are in red. Some branches were shortened according to the indicated multipliers.

Three new strains of *Helminthosporium* isolated from dead branches in Xishuangbanna, Yunnan Province, China, were grown in culture and used for analyses of molecular sequence data. ​Unfortunately, our two species, *H.nabanhensis* and *H.sinensis* lack the *RPB2* sequences. Newly generated sequences were deposited in GenBank. Alignment has 75 sequences with 1511 total characters (The combined dataset, ITS:1–457, LSU:458–993, *RBP2*:994–1110, SSU:1111–1363, *TEF1*:1364–1511), 555 distinct patterns, 487 parsimony-informative, 89 singleton sites, 935 constant sites, and *Cyclothyriellarubronotata* (TR) and *C.rubronotata* (TR9) were regarded as an outgroup. Maximum likelihood and Bayesian Inference analyses of the combined dataset resulted in phylogenetic reconstructions with largely similar topologies, and bootstrap support values for Maximum likelihood higher than 90% and Bayesian posterior probabilities greater than 0.90 are given above the nodes. The best-scoring ML consensus tree (lnL = –10,686.191) with ultrafast bootstrap values from ML analyses and posterior probabilities from MrBayes analysis at the nodes are shown in Fig. 1. *Helminthosporiumnabanhensis* form a distinct clade sister to *H.chlorophorae* with strong statistical support (ML/BI = 95/1.00); *H.sinensis* forms a high-support clade (ML/BI = 92/0.99) with the lineage consisting of *H.nabanhensis* and *H.chlorophorae*; *H.yunnanensis* is a sister to three different strains of *H.austriacum* with strong statistical support (ML/BI = 100/1.00).

### ﻿Taxonomy

#### ﻿ Helminthosporium nabanhensis

Taxon classificationFungiPleosporalesMassarinaceae

Jing W. Liu & Jian Ma sp. nov.

02A0416F-9863-51D6-8336-453546040DBD

IndexFungorum No: 559980

Fig. 2

##### Etymology.

Referring to the collecting site of Nabanhe Nature Reserve in Yunnan Province, China.

##### Holotypus.

HJAUP M2054.

##### Description.

Saprobic on dead branches. ***Sexual morph***: Undetermined. ***Asexual morph***: Hyphomycetous. ***Colonies*** on natural substrate effuse, scattered, hairy, brown to black. ***Mycelium*** partly superficial, partly immersed in the substratum, composed of branched, septate, pale brown to brown, smooth hyphae. ***Conidiophores*** macronematous, mononematous, solitary or in groups of 2–4, simple, occasionally branched, erect, straight or flexuous, cylindrical, smooth, 8–21-septate, brown to dark brown, paler towards the apex, with well-defined small pores at the apex and rarely laterally beneath the upper 1–3 septa, 365–557 × 6.5–13.5 μm. ***Conidiogenous cells*** polytretic, integrated, terminal and intercalary, cylindrical, brown, smooth, with noncicatrized, distinct pores. Conidial secession schizolytic. ***Conidia*** acropleurogenous, solitary, dry, obclavate, pale brown to brown, 3–6-distoseptate, smooth, straight or curved, wider below than apex, truncate and dark at base, apically rostrate and pale, guttulate when young, non-guttulate at maturity, 26.5–46.5 μm long, 6.5–10 μm wide, tapering to 3–3.5 μm wide near the apex, 3–6 μm wide at the basal scar.

**Figure 2.**
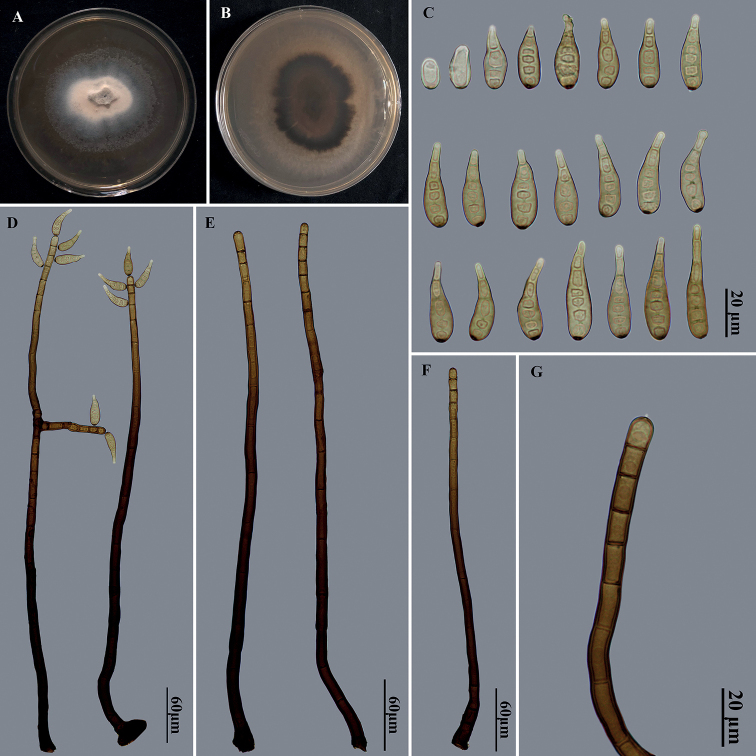
*Helminthosporiumnabanhensis* (HJAUPM2054, holotype) **A, B** culture on PDA from above and reverse **C** conidia **D** conidiophores with conidia **E–G** conidiophores with conidiogenous cells.

##### Cultural characteristics.

Colony on PDA reaching 50–55 mm diam. after 2 weeks in an incubator under dark conditions at 25 °C, irregular circular, surface velvety, with white and denser mycelium at the center, becoming olivaceous and sparser towards the edge; reverse pale brown at the center, dark brown at the periphery.

##### Material examined.

China, Yunnan Province: Xishuangbanna Dai Autonomous Prefecture, Nabanhe National Nature Reserve, on dead branches of an unidentified broadleaf tree, 12 July 2021, J.W. Liu, HJAUP M2054 (Holotype), ex-type living culture HJAUP C2054.

##### Notes.

The phylogenetic tree shows that the strain of *H.nabanhensis* (HJAUP C2054) clusters with the ex-type strain of *H.chlorophorae* (BRIP 14521). The BLASTn analysis of ITS of our ex-type strain HJAUP C2054 showed 90% identity (425/471 bp, 10/471 gaps) with ex-type strain BRIP 14521 of *H.chlorophorae*. Moreover, *H.nabanhensis* morphologically differs from *H.chlorophorae* in bigger conidiophores (365–557 × 6.5–13.5 μm vs. 120–270 × 7–10 μm) occasionally branched, and smaller conidia (26.5–46.5 × 6.5–10 μm vs. 52–102 × 8–11 μm) with fewer septa (3–6 vs. 6–9), and from *H.sichuanense* (Zhang et al. 2004) in narrower conidiophores (6.5–13.5 μm vs. 14–25 μm) and smaller conidia (26.5–46.5 × 6.5–10 μm vs. 41–86 × 10–14 μm) with fewer septa (3–6 vs. 5–11).

#### ﻿ Helminthosporium sinensis

Taxon classificationFungiPleosporalesMassarinaceae

Jing W. Liu & Jian Ma sp. nov.

36669D25-D9A2-502E-9F90-85C0EBE6CC01

IndexFungorum No: 559981

Fig. 3

##### Etymology.

Referring to the country in which the fungus was collected.

##### Holotypus.

HJAUP M2121.

##### Description.

Saprobic on dead branches. ***Sexual morph***: Undetermined. ***Asexual morph***: Hyphomycetous. ***Colonies*** on natural substrate effuse, scattered, hairy, brown to black. ***Mycelium*** partly superficial, partly immersed in the substratum, composed of branched, septate, pale brown to brown, smooth hyphae. ***Conidiophores*** macronematous, mononematous, solitary or in groups of 2–4, simple, straight or flexuous, thick-walled, cylindrical, smooth, brown to dark brown, paler towards the apex, with well-defined small pores at the apex and rarely laterally beneath the upper 1–4 septa, 220–370 × 6–8.5 μm. ***Conidiogenous cells*** polytretic, integrated, terminal and intercalary, cylindrical, brown, smooth, with noncicatrized, distinct pores. Conidial secession schizolytic. ***Conidia*** acropleurogenous, solitary, rarely catenate, dry, obclavate, pale brown, 2–7-distoseptate, smooth, straight or curved, wider below than apex, truncate and dark at base, apically rostrate and pale, 37–60 μm long, 5.5–8.5 μm wide, tapering to 3–3.5 μm wide near the apex, 3–6 μm wide at the basal scar.

**Figure 3.**
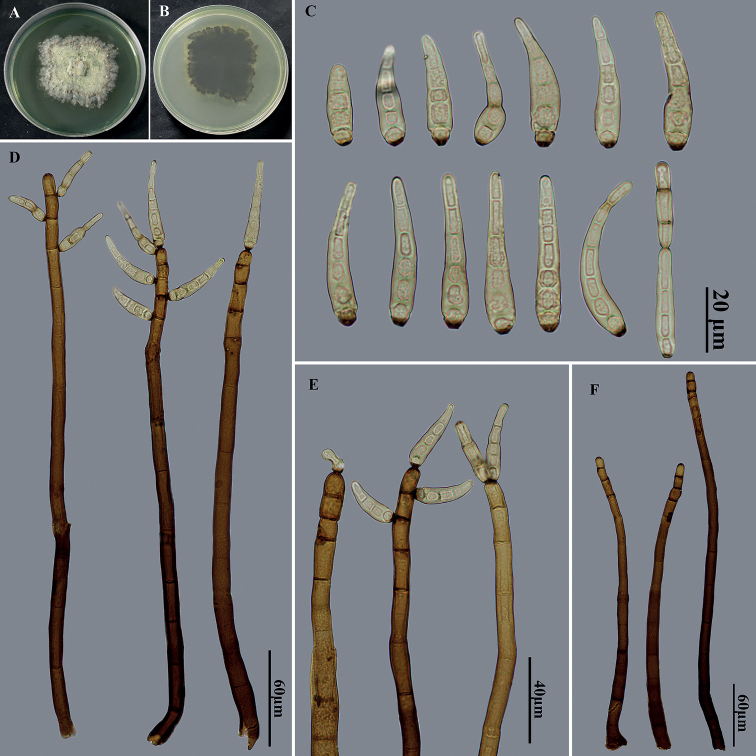
*Helminthosporiumsinensis* (HJAUPM2121, holotype) **A, B** culture on PDA from above and reverse **C** conidia **D, E** conidiophores, conidiogenous cells and conidia **F** conidiophores.

##### Cultural characteristics.

Colony on PDA reaching 30–37 mm diam. after 2 weeks in an incubator under dark conditions at 25 °C, pale brown, irregular circular, surface velvety, outermost layer gray; reverse dark brown, produces pale green pigment.

##### Material examined.

China, Yunnan Province: Xishuangbanna Dai Autonomous Prefecture, Menghai County, Mengsong Township, on dead branches of an unidentified broadleaf tree, 13 July 2021, J.W. Liu, HJAUP M2121 (Holotype), ex-type living culture HJAUP C2121.

##### Notes.

Phylogenetic analysis shows that the strain of *H.sinensis* (HJAUP C2121) forms an independent clade, and clusters with the strains of *H.nabanhensis* (HJAUP C2054) and *H.chlorophorae* (BRIP 14521). The BLASTn analysis of ITS of our ex-type strain HJAUP C2121 showed 89% identity (536/602 bp, 17/602 gaps) with ex-type strain HJAUP C2054 of *H.nabanhensis*, and showed 91% identity (430/471 bp, 13/471 gaps) with ex-type strain BRIP 14521 of *H.chlorophorae*. Moreover, *H.sinensis* differs from *H.nabanhensis* by its longer and narrower conidia (37–60 × 5.5–8.5 μm vs. 26.5–46.5 × 6.5–10 μm), and smaller conidiophores (220–370 × 6–8.5 μm vs. 365–557 × 6.5–13.5 μm), and from *H.chlorophorae* by its smaller conidia (37–60 × 5.5–8.5 μm vs. 52–102 × 8–11 μm) and longer and narrower conidiophores (220–370 × 6–8.5 μm vs. 120–270 × 7–10 μm), and from *H.guangxiense* (Zhang and Zhang 2009) in smaller conidiophores (220–370 × 6–8.5 μm vs. 330–850 × 14–25 μm) and smaller conidia (37–60 × 5.5–8.5 μm vs. 76–110 × 16–22 μm) with fewer septa (2–7 vs. 9–17). In addition, the conidia of *H.sinensis* are solitary or rarely catenate, whereas those of *H.guangxiense*, *H.nabanhensis* and *H.chlorophorae* are solitary.

#### ﻿ Helminthosporium yunnanensis

Taxon classificationFungiPleosporalesMassarinaceae

Jing W. Liu & Jian Ma sp. nov.

A5E3A1DD-124B-5161-B78A-9AAA9B5EFA2D

IndexFungorum No: 559982

Fig. 4

##### Etymology.

Referring to Yunnan province, where the type specimen was collected.

##### Holotypus.

HJAUP M2071.

##### Description.

Saprobic on dead branches. ***Sexual morph***: Undetermined. ***Asexual morph***: Hyphomycetous. ***Colonies*** on natural substrate effuse, scattered, hairy, brown to dark brown. Mycelium partly superficial, partly immersed in the substratum, composed of branched, septate, pale brown to brown, smooth hyphae. ***Conidiophores*** macronematous, mononematous, solitary or in groups of 2–4, simple, straight or flexuous, thick-walled, cylindrical, smooth, brown to dark brown, paler towards the apex, with one cylindrical, enteroblastic percurrent extension, and with well-defined small pores at the apex and rarely laterally beneath the upper 1–5 septa, 560–680 × 12.5–15.5 μm. ***Conidiogenous cells*** polytretic, integrated, terminal and intercalary, cylindrical, pale brown to brown, smooth, with noncicatrized, distinct pores. Conidial secession schizolytic. ***Conidia*** acropleurogenous, solitary, dry, obclavate, sigmoid, lunate or uncinate, pale brown, 4–7-distoseptate, smooth, straight or flexuous, wider below than apex, truncate and dark at base, apically rostrate and pale, 30.5–55.5 μm long, 9–11 μm wide, tapering to 2.5–3 μm near the apex, 3–7.5 μm wide at the basal scar.

**Figure 4.**
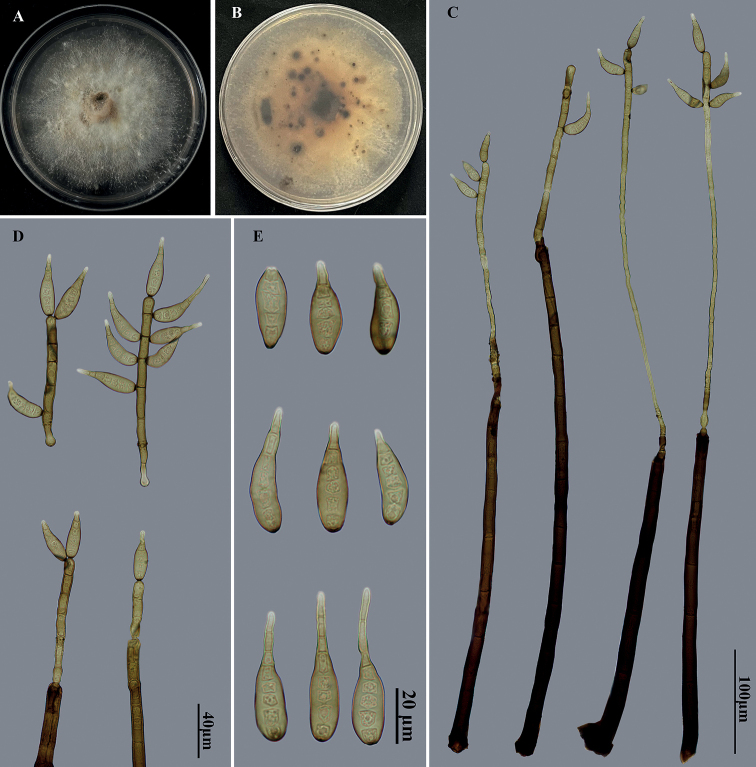
*Helminthosporiumyunnanensis* (HJAUPM2071, holotype) **A, B** culture on PDA from above and reverse **C** conidiophores with conidia **D** conidiogenous cells and conidia **E** conidia.

##### Cultural characteristics.

Colony on PDA reaching 75–82 mm diam. after 2 weeks in an incubator under dark conditions at 25 °C, irregular circular, surface velvety, with brown and denser mycelium at the center, becoming white and sparser towards the edge; reverse pale brown at the center, with little black dots.

##### Material examined.

China, Yunnan Province: Xishuangbanna Dai Autonomous Prefecture, Nabanhe National Nature Reserve, on dead branches of an unidentified broadleaf tree, 12 July 2021, J.W. Liu, HJAUP M2071 (Holotype), ex-type living culture HJAUP C2071.

##### Notes.

Phylogenetic analysis shows that the strain of *H.yunnanensis* (HJAUP C2071) clustered together and formed a sister clade with three different strains of *H.austriacum* (L132, L137, L169) (Voglmayr and Jaklitsch 2017). The BLASTn analysis of *H.yunnanensis* (HJAUP C2071) and *H.austriacum* (L132^HT^) shows 97% identity (524/541, 4 gaps) using ITS, 99% identity (550/553, 2 gaps) using LSU, 99% identity (872/873, 1 gap) using SSU, 98% identity (738/752, no gap) using *TEF1*, and 98% identity (1077/1095, no gap) using *RPB2*. *Helminthosporiumyunnanensis* morphologically differs from *H.austriacum* in wider conidiophores (560–680 × 12.5–15.5 μm vs. 275–700 × 7–11 μm) with one cylindrical, enteroblastic percurrent extension, and narrower conidia (30.5–55.5 × 9–11 μm vs. 35–48 × 13.7–16.5 μm), and from *H.obpyriforme* (Zhang and Zhang 2009) in bigger conidiophores (560–680 × 12.5–15.5 μm vs. 225–460 × 9.5–13 μm) and smaller conidia (30.5–55.5 × 9–11 μm vs. 47–74 × 14–19 μm) with fewer septa (4–7 vs. 5–9).

## ﻿Discussion

The taxonomic history of the genus *Helminthosporium* is complex. To date, about 770 epithets for *Helminthosporium* are listed in Index Fungorum (2022), but most of these were not congeneric with the generic type. Konta et al. (2021) listed 216 *Helminthosporium* species based on records from Species Fungorum, but most species are identified based on morphological studies, and so far only 27 species are represented by a DNA sequence in GenBank (Voglmayr and Jaklitsch 2017; Boonmee et al. 2021; Chen et al. 2022). Morphological comparison is important for fungal identification, but species identification only based on morphological studies is not comprehensive. With the availability of supplementary sequence data for *Helminthosporium* species, the molecular phylogenetic analysis is being used to evaluate previously described *Helminthosporium*-like species by molecular methods. The introduction of a phylogenetic analysis of *Helminthosporium* led to a better improvement of the heterogeneity of the genus and further clarified the taxonomic status of *Helminthosporium*. Voglmayr and Jaklitsch (2017) revisited *Corynespora*, *Exosporium* and *Helminthosporium*, with phylogenetic and morphological analyses. Zhang et al. (2020b) transferred *H.bigenum* into a new genus *Mirohelminthosporium* K. Zhang, D.W. Li & R.F. Castañeda and replaced the illegitimate *H.cylindrosporum* Matsush. with *H.matsushimae*. Chen et al. (2022) suggested four *Helminthosporium* species, *H.anomalum*, *H.asterinum*, *H.decacuminatum* and *H.gibberosporum* to *Bipolaris*, *Kirschsteiniothelia* or *Curvularia* by performing blastn analysis. ​Furthermore, seven new species were described under the genus *Helminthosporium* by molecular methods (Crous et al. 2018, 2019; Zhao et al. 2018; Boonmee et al. 2021; Chen et al. 2022). Based on previous studies, we proposed three new species by morphological and molecular phylogenetic analysis.

Chen et al. (2022) described two new species, *H.chengduense* and *H.chinense*, based on combined ITS, LSU, SSU, *TEF1* and *RPB2* sequence data and morphological characters. Accordingly, we also used ITS, LSU, SSU, *TEF1* and *RPB2* for phylogenetic analysis and obtained high phylogenetic support, although our two species, *H.nabanhensis* and *H.sinensis*, lack the *RPB2* sequences. They are considerably distinct from all other described *Helminthosporium* species by morphological characters and multi-locus phylogenetic analysis, so we are convinced that the newly introduced species are new to science.

## Supplementary Material

XML Treatment for Helminthosporium nabanhensis

XML Treatment for Helminthosporium sinensis

XML Treatment for Helminthosporium yunnanensis
